# Supplementary material for: Hyperbolic band topology with non-trivial second Chern numbers
Source: Nat Commun. 2023 Feb 25;14:1083. doi: 10.1038/s41467-023-36767-8 (PMC9968300; doi:10.1038/s41467-023-36767-8)
Supplement: Supplementary file 1 — Supplementary Information [file 41467_2023_36767_MOESM1_ESM.pdf]

Supplementary Information: Hyperbolic band topology with non-trivial second Chern numbers

W. Zhang et al.

Supplementary Note 1. The deviation of hyperbolic Hamiltonian in the momentum space.

Supplementary Note 2. Periodic boundary conditions of the abelian and non-abelian clusters.

Supplementary Note 3. The $U(1)$ hyperbolic band theory of the abelian cluster with non-trivial topologies.

Supplementary Note 4. Numerical results of the eigen-spectra for non-abelian clusters with PBCs.

Supplementary Note 5. Numerical results of the eigen-spectra for abelian clusters with fully OBCs.

Supplementary Note 6. Details for the partially OBCs of abelian clusters.

Supplementary Note 7. Details for the derivation of eigenequations for hyperbolic circuits.

Supplementary Note 8. The influence of lossy effects on impedance responses.

Supplementary Note 1. The deviation of hyperbolic Hamiltonian in the momentum space. In this part, we give a detailed deviation of hyperbolic Hamiltonian in the momentum space. Our model in the real space can be described by the following tight-binding Hamiltonian

$$\begin{aligned}H = \sum_{i,j,k,l=1}^{n_1,n_2,n_3,n_4} [(&-0.5it_2 d^+_{i,j+1,k,l}a_{i,j,k,l} - 0.5t_3 d^+_{i,j,k+1,l}a_{i,j,k,l} + 0.5t_1 c^+_{i+1,j,k,l}a_{i,j,k,l} - 0.5it_4 c^+_{i,j,k,l+1}a_{i,j,k,l}\\
&-0.5it_2 c^+_{i,j+1,k,l}b_{i,j,k,l} + 0.5t_3 c^+_{i,j,k+1,l}b_{i,j,k,l} + 0.5t_1 d^+_{i+1,j,k,l}b_{i,j,k,l} + 0.5it_4 d^+_{i,j,k,l+1}b_{i,j,k,l}\\
&-0.5it_2 b^+_{i,j+1,k,l}c_{i,j,k,l} - 0.5t_3 b^+_{i,j,k+1,l}c_{i,j,k,l} - 0.5t_1 a^+_{i+1,j,k,l}c_{i,j,k,l} - 0.5it_4 a^+_{i,j,k,l+1}c_{i,j,k,l}\\
&-0.5it_2 a^+_{i,j+1,k,l}d_{i,j,k,l} + 0.5t_3 a^+_{i,j,k+1,l}d_{i,j,k,l} - 0.5t_1 b^+_{i+1,j,k,l}d_{i,j,k,l} + 0.5it_4 b^+_{i,j,k,l+1}d_{i,j,k,l}\\
&+0.5J_1 a^+_{i+1,j,k,l}a_{i,j,k,l} + 0.5J_2 a^+_{i,j+1,k,l}a_{i,j,k,l} + 0.5J_3 a^+_{i,j,k+1,l}a_{i,j,k,l} + 0.5J_4 a^+_{i,j,k,l+1}a_{i,j,k,l}\\
&+0.5J_1 b^+_{i+1,j,k,l}b_{i,j,k,l} + 0.5J_2 b^+_{i,j+1,k,l}b_{i,j,k,l} + 0.5J_3 b^+_{i,j,k+1,l}b_{i,j,k,l} + 0.5J_4 b^+_{i,j,k,l+1}b_{i,j,k,l}\\
&-0.5J_1 c^+_{i+1,j,k,l}c_{i,j,k,l} - 0.5J_2 c^+_{i,j+1,k,l}c_{i,j,k,l} - 0.5J_3 c^+_{i,j,k+1,l}c_{i,j,k,l} - 0.5J_4 c^+_{i,j,k,l+1}c_{i,j,k,l}\\
&-0.5J_1 d^+_{i+1,j,k,l}d_{i,j,k,l} - 0.5J_2 d^+_{i,j+1,k,l}d_{i,j,k,l} - 0.5J_3 d^+_{i,j,k+1,l}d_{i,j,k,l} - 0.5J_4 d^+_{i,j,k,l+1}d_{i,j,k,l} + h.c.)\\
&+(m-a)a^+_{i,j,k,l}a_{i,j,k,l} + (m+a)b^+_{i,j,k,l}b_{i,j,k,l} + (-m-a)c^+_{i,j,k,l}c_{i,j,k,l} + (-m+a)d^+_{i,j,k,l}d_{i,j,k,l}]\end{aligned} \quad (1)$$

Here, $a_{i,j,k,l}$ ($a^+_{i,j,k,l}$), $b_{i,j,k,l}$ ($b^+_{i,j,k,l}$), $c_{i,j,k,l}$ ($c^+_{i,j,k,l}$), and $d_{i,j,k,l}$ ($d^+_{i,j,k,l}$) correspond to annihilation (creation) operators of four sublattices in the unit cell marked by (*i, j, k, l*), which quantify the implementation times of group operators $\gamma_1, \gamma_2, \gamma_3, \gamma_4$ on the central unit.

n_1, n_2, n_3 and n_4 represent the number of unit cells along the translational directions related to $\gamma_1, \gamma_2, \gamma_3, \gamma_4$, respectively. Onsite potentials of four sublattices labeled by $a_{i,j,k,l}$ ($a^+_{i,j,k,l}$), $b_{i,j,k,l}$ ($b^+_{i,j,k,l}$), $c_{i,j,k,l}$ ($c^+_{i,j,k,l}$), and $d_{i,j,k,l}$ ($d^+_{i,j,k,l}$) equal to *m-a, m+a, -m+a, -m-a,* respectively. The inter-cell coupling strengths along the hyperbolic translation direction marked by γ_j are described by $\pm J_j$ (with *j*=1, 2, 3, 4), $\pm t_j$ (with *j*=1, 3) and $\pm it_j$ (with *j*= 2, 4), respectively. Following the hyperbolic band theory, the above lattice model in 2D hyperbolic space can be described in the momentum space. In particular, we can equip inter-cell couplings of the hyperbolic unit cell with twisted boundary conditions along four translation directions, where the phase factors e^{ik_j} (*j*=1, 2, 3, 4) along directions given by $\gamma_1, \gamma_2, \gamma_3$ and γ_4 are introduced. The phase factors related to their inverses are in the form of e^{-ik_j} (*j*=1, 2, 3, 4). In this case, four wave-vectors k_1, k_2, k_3 and k_4 can be regarded as Bloch vectors in 4D momentum space.

To get the hyperbolic Hamiltonian in momentum space, we apply the Fourier transform of the real space Hamiltonian by expressing the annihilation (creation) operators of four sublattices in the *k*-space as

$$\begin{aligned}
a_{i,j,k,l} &= \frac{1}{\sqrt{n_1n_2n_3n_4}}\sum_{i=1}^{n_4}\sum_{j=1}^{n_3}\sum_{k=1}^{n_2}\sum_{l=1}^{n_1} e^{-i(ik_1+jk_2+kk_3+lk_4)}a_{k_1,k_2,k_3,k_4}\\
a^+_{i,j,k,l} &= \frac{1}{\sqrt{n_1n_2n_3n_4}}\sum_{i=1}^{n_4}\sum_{j=1}^{n_3}\sum_{k=1}^{n_2}\sum_{l=1}^{n_1} e^{i(ik_1+jk_2+kk_3+lk_4)}a^+_{k_1,k_2,k_3,k_4}\\
b_{i,j,k,l} &= \frac{1}{\sqrt{n_1n_2n_3n_4}}\sum_{i=1}^{n_4}\sum_{j=1}^{n_3}\sum_{k=1}^{n_2}\sum_{l=1}^{n_1} e^{-i(ik_1+jk_2+kk_3+lk_4)}b_{k_1,k_2,k_3,k_4}\\
b^+_{i,j,k,l} &= \frac{1}{\sqrt{n_1n_2n_3n_4}}\sum_{i=1}^{n_4}\sum_{j=1}^{n_3}\sum_{k=1}^{n_2}\sum_{l=1}^{n_1} e^{i(ik_1+jk_2+kk_3+lk_4)}b^+_{k_1,k_2,k_3,k_4}\\
c_{i,j,k,l} &= \frac{1}{\sqrt{n_1n_2n_3n_4}}\sum_{i=1}^{n_4}\sum_{j=1}^{n_3}\sum_{k=1}^{n_2}\sum_{l=1}^{n_1} e^{-i(ik_1+jk_2+kk_3+lk_4)}c_{k_1,k_2,k_3,k_4}\\
c^+_{i,j,k,l} &= \frac{1}{\sqrt{n_1n_2n_3n_4}}\sum_{i=1}^{n_4}\sum_{j=1}^{n_3}\sum_{k=1}^{n_2}\sum_{l=1}^{n_1} e^{i(ik_1+jk_2+kk_3+lk_4)}c^+_{k_1,k_2,k_3,k_4}\\
d_{i,j,k,l} &= \frac{1}{\sqrt{n_1n_2n_3n_4}}\sum_{i=1}^{n_4}\sum_{j=1}^{n_3}\sum_{k=1}^{n_2}\sum_{l=1}^{n_1} e^{-i(ik_1+jk_2+kk_3+lk_4)}d_{k_1,k_2,k_3,k_4}\\
d^+_{i,j,k,l} &= \frac{1}{\sqrt{n_1n_2n_3n_4}}\sum_{i=1}^{n_4}\sum_{j=1}^{n_3}\sum_{k=1}^{n_2}\sum_{l=1}^{n_1} e^{i(ik_1+jk_2+kk_3+lk_4)}d^+_{k_1,k_2,k_3,k_4}
\end{aligned} \tag{2}$$

Here, a_{k_1,k_2,k_3,k_4} ($a^+_{k_1,k_2,k_3,k_4}$), b_{k_1,k_2,k_3,k_4} ($b^+_{k_1,k_2,k_3,k_4}$), c_{k_1,k_2,k_3,k_4} ($c^+_{k_1,k_2,k_3,k_4}$), and d_{k_1,k_2,k_3,k_4} ($d^+_{k_1,k_2,k_3,k_4}$) correspond to annihilation (creation) operators of four sublattices in *k*-space marked by $k_1, k_2, k_3, and\ k_4$. Submitting Eq. (2) in to Eq. (1), the k-space Hamiltonian can be written as

$$\begin{aligned}
H(k) = \frac{1}{n_1n_2n_3n_4}\sum_{i,j,k,l=1}^{n_1,n_2,n_3,n_4} &[t_2 sin(k_2) d^+_{k_1,k_2,k_3,k_4}a_{k_1,k_2,k_3,k_4} - it_3 sin(k_3) d^+_{k_1,k_2,k_3,k_4}a_{k_1,k_2,k_3,k_4}\\
&+it_1 sin(k_1) c^+_{k_1,k_2,k_3,k_4}a_{k_1,k_2,k_3,k_4} + t_4 sin(k_4) c^+_{k_1,k_2,k_3,k_4}a_{k_1,k_2,k_3,k_4}\\
&+t_2 sin(k_2) c^+_{k_1,k_2,k_3,k_4}b_{k_1,k_2,k_3,k_4} + it_3 sin(k_3) c^+_{k_1,k_2,k_3,k_4}b_{k_1,k_2,k_3,k_4}\\
&+it_1 sin(k_1) d^+_{k_1,k_2,k_3,k_4}b_{k_1,k_2,k_3,k_4} - t_4 sin(k_4) d^+_{k_1,k_2,k_3,k_4}b_{k_1,k_2,k_3,k_4}\\
&+t_2 sin(k_2) b^+_{k_1,k_2,k_3,k_4}c_{k_1,k_2,k_3,k_4} - it_3 sin(k_3) b^+_{k_1,k_2,k_3,k_4}c_{k_1,k_2,k_3,k_4}\\
&-it_1 sin(k_1) a^+_{k_1,k_2,k_3,k_4}c_{k_1,k_2,k_3,k_4} + t_4 sin(k_4) a^+_{k_1,k_2,k_3,k_4}c_{k_1,k_2,k_3,k_4}\\
&+t_2 sin(k_2) a^+_{k_1,k_2,k_3,k_4}d_{k_1,k_2,k_3,k_4} + it_3 sin(k_3) a^+_{k_1,k_2,k_3,k_4}d_{k_1,k_2,k_3,k_4}
\end{aligned}$$

$$-it_1 sin(k_1) b^+_{k_1,k_2,k_3,k_4} d_{k_1,k_2,k_3,k_4} - t_4 sin(k_4) b^+_{k_1,k_2,k_3,k_4} d_{k_1,k_2,k_3,k_4}$$

$$+J_1 cos(k_1) a^+_{k_1,k_2,k_3,k_4} a_{k_1,k_2,k_3,k_4} + J_2 cos(k_2) a^+_{k_1,k_2,k_3,k_4} a_{k_1,k_2,k_3,k_4}$$

$$+J_3 cos(k_3) a^+_{k_1,k_2,k_3,k_4} a_{k_1,k_2,k_3,k_4} + J_4 cos(k_4) a^+_{k_1,k_2,k_3,k_4} a_{k_1,k_2,k_3,k_4}$$

$$+J_1 cos(k_1) b^+_{k_1,k_2,k_3,k_4} b_{k_1,k_2,k_3,k_4} + J_2 cos(k_2) b^+_{k_1,k_2,k_3,k_4} b_{k_1,k_2,k_3,k_4}$$

$$+J_3 cos(k_3) b^+_{k_1,k_2,k_3,k_4} b_{k_1,k_2,k_3,k_4} + J_4 cos(k_4) b^+_{k_1,k_2,k_3,k_4} b_{k_1,k_2,k_3,k_4}$$

$$-J_1 cos(k_1) c^+_{k_1,k_2,k_3,k_4} c_{k_1,k_2,k_3,k_4} - J_2 cos(k_2) c^+_{k_1,k_2,k_3,k_4} c_{k_1,k_2,k_3,k_4}$$

$$-J_3 cos(k_3) c^+_{k_1,k_2,k_3,k_4} c_{k_1,k_2,k_3,k_4} - J_4 cos(k_4) c^+_{k_1,k_2,k_3,k_4} c_{k_1,k_2,k_3,k_4}$$

$$-J_1 cos(k_1) d^+_{k_1,k_2,k_3,k_4} d_{k_1,k_2,k_3,k_4} - J_2 cos(k_2) d^+_{k_1,k_2,k_3,k_4} d_{k_1,k_2,k_3,k_4}$$

$$-J_3 cos(k_3) d^+_{k_1,k_2,k_3,k_4} d_{k_1,k_2,k_3,k_4} - J_4 cos(k_4) d^+_{k_1,k_2,k_3,k_4} d_{k_1,k_2,k_3,k_4}]$$

$$+(m-a) a^+_{k_1,k_2,k_3,k_4} a_{k_1,k_2,k_3,k_4} + (m+a) b^+_{k_1,k_2,k_3,k_4} b_{k_1,k_2,k_3,k_4}$$

$$+(-m+a) c^+_{k_1,k_2,k_3,k_4} c_{k_1,k_2,k_3,k_4} + (-m-a) d^+_{k_1,k_2,k_3,k_4} d_{k_1,k_2,k_3,k_4}] \quad (3)$$

In this case, we can re-express the k-space Hamilton in the matrix form of

$$H = \boldsymbol{d}(\boldsymbol{k}) \cdot \boldsymbol{\Gamma} + ia\Gamma_1\Gamma_4, \quad (4)$$

where the vector $\boldsymbol{d}(\boldsymbol{k})$ is in the form of $\boldsymbol{d}(\boldsymbol{k}) = \{t_1 sin(k_1), t_2 sin(k_2), t_3 sin(k_3), t_4 sin(k_4), m + \sum_{j=1}^{4} J_j \cos(k_j)\}$ and the vector of gamma matrices $\boldsymbol{\Gamma} = \{\Gamma_1, \Gamma_2, \Gamma_3, \Gamma_4, \Gamma_5\}$ satisfies the Clifford algebra. Detailed expressions of these matrices are written as $\Gamma_1 = -\sigma_2 \otimes I$, $\Gamma_2 = \sigma_1 \otimes \sigma_1$, $\Gamma_3 = \sigma_1 \otimes \sigma_2$, $\Gamma_4 = \sigma_1 \otimes \sigma_3$ and $\Gamma_5 = \sigma_3 \otimes I$. I is the 2 by 2 identity matrix and σ_j (j=1, 2, 3) are Pauli matrices.

Supplementary Note 2. Periodic boundary conditions of the abelian and non-abelian clusters.

In this part, we illustrate the detailed boundary connection to construct the PBC for both abelian and non-abelian clusters. Before the discussion on finite hyperbolic clusters, we start to illustrate the general process to produce our designed hyperbolic lattice model. Based on the crystallography of {8,8} hyperbolic lattices, it has been pointed out that the {8, 8} hyperbolic lattice can be constructed by applying translational operations (generated by four generators of $\gamma_1, \gamma_2, \gamma_3$ and γ_4) to the unit cell. The representation of these generators in the Poincaré disk can be expressed as $\gamma_u = R((u-1)\alpha_B)\gamma_1 R(-(u-1)\alpha_B)$ with $u = 1,2,3,4$. Here, R is the rotation matrix written by $R((u-1)\alpha_B) = \begin{pmatrix} \exp(i(u-1)\alpha_B/2) & \sigma \\ \sigma & \exp(i(u-1)\alpha_B/2) \end{pmatrix}$ with $\alpha_B = 2\pi/8$. γ_1 is the first generator described by $\gamma_1 = \frac{1}{\sqrt{1-\sigma^2}}\begin{pmatrix} 1 & \sigma \\ \sigma & 1 \end{pmatrix}$ with $\sigma = \sqrt{(cos(\alpha_B) + cos(\beta_B))/(1 + cos(\beta_B))}$ and $\beta_B = 2\pi/8$. By using these generators, the hyperbolic model can be created. As shown in the left inset of Supplementary Figure 1a, we start to apply eight translational operations $(\gamma_1, \gamma_2, \gamma_3, \gamma_4, \gamma_1^{-1}, \gamma_2^{-1}, \gamma_3^{-1}, \gamma_4^{-1})$ to the central unit with four sublattices, which are enclosed by the

black dash block. These operations on the central unit can generate eight first-generation units. The inter-cell couplings along these directions (not shown here) are illustrated in Fig. 1b of main text. Then, we apply eight translational operations to each first-generation unit (enclosed by red dash blocks), as shown in the right inset of Supplementary Figure 1a. It is noted that there are eight second-generation units being coincident to the central unit. In this case, we have totally 56 second-generation units. Repeating the above process, we can obtain the hyperbolic lattice model with high-generation units.

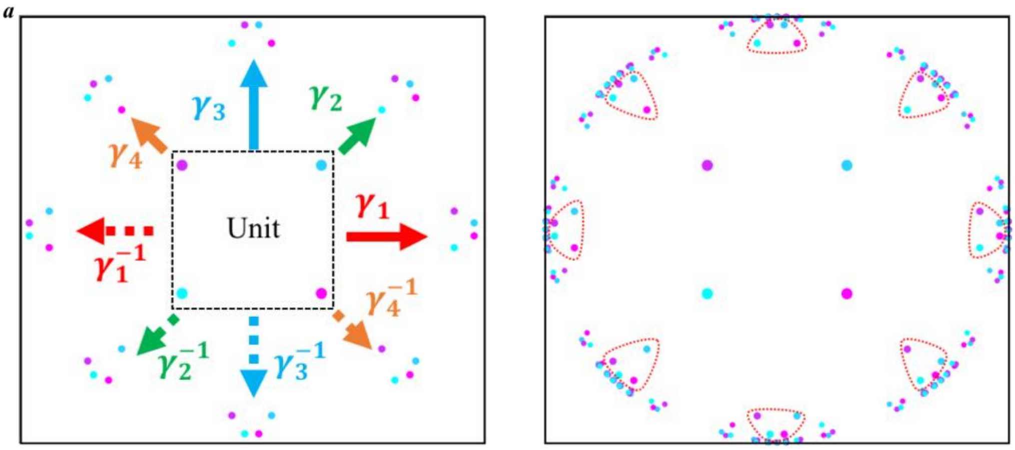

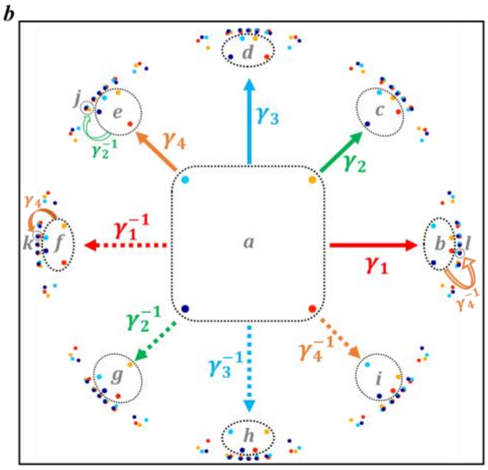

c

Abelian cluster

γ_1	e to g	j to d	f to a	k to e	g to k	h to j	i to l	l to c
γ_2	e to f	j to e	f to l	k to b	g to a	h to k	i to j	l to d
γ_3	e to i	j to b	f to j	k to c	g to l	h to a	i to k	l to e
γ_4	e to h	j to c	f to k	k to d	g to j	h to l	i to a	l to b
γ_1^{-1}	e to k	j to h	f to b	k to g	g to e	h to d	i to c	l to i
γ_2^{-1}	e to j	j to i	f to e	k to h	g to d	h to c	i to b	l to f
γ_3^{-1}	e to l	j to f	f to d	k to i	g to c	h to b	i to e	l to g
γ_4^{-1}	e to a	j to g	f to c	k to f	g to b	h to e	i to d	l to h

d

Non-abelian cluster

γ_1	e to h	j to d	f to a	k to c	g to j	h to l	i to k	l to e
γ_2	e to f	j to e	f to j	k to b	g to a	h to k	i to l	l to d
γ_3	e to g	j to b	f to l	k to e	g to k	h to a	i to j	l to c
γ_4	e to i	j to c	f to k	k to d	g to l	h to j	i to a	l to b
γ_1^{-1}	e to l	j to g	f to b	k to i	g to d	h to e	i to c	l to h
γ_2^{-1}	e to j	j to f	f to e	k to h	g to c	h to b	i to d	l to i
γ_3^{-1}	e to k	j to i	f to c	k to g	g to e	h to d	i to b	l to f
γ_4^{-1}	e to a	j to h	f to d	k to f	g to b	h to c	i to e	l to g

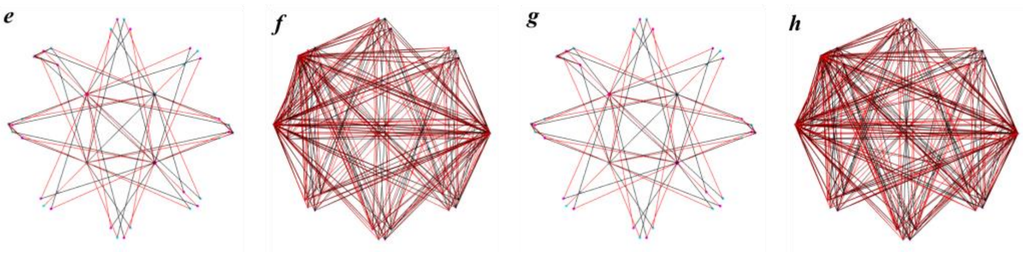

Supplementary Figure 1. Periodic boundary conditions of the abelian and non-abelian clusters. (a) The illustration of generating process for the hyperbolic lattice model. (b). Twelve units marked by letters ranging from *a* to *l* in the finite lattice of hyperbolic plane. (c) and (d) The associated generator to generate the inter-cell couplings of boundary units along eight directions for the abelian

and non-abelian clusters, respectively. (e) and (f) The bulk and boundary connection patterns of the abelian cluster. (g) and (h) The bulk and boundary connection patterns of the non-abelian cluster. The red and black lines correspond to the couplings related to t_j and J_j, respectively.

Then, we turn to the hyperbolic cluster with a finite number of units. As shown in Supplementary Figure 1b, we label 12 units of our considered finite lattice by 12 letters ranging from *a* to *l*. Supplementary Figures 1c and 1d present the associated generator to generate the inter-cell couplings of boundary units along eight directions for the abelian and non-abelian clusters, respectively. In this case, we can see that the boundary unit possesses inter-cell couplings along eight different directions, and the number of inter-site connection of all sites equals to 16, manifesting the effectiveness of the applied PBCs. To further clarify the connection patterns, in Supplementary Figures 1e and 1f (Supplementary Figures 1g and 1h), we plot the bulk and boundary connection patterns of the abelian (non-abelian) cluster. The red and black lines correspond to the couplings related to t_j and J_j, respectively. Specifically, the bulk-connection pattern corresponds to the finite hyperbolic cluster under OBCs, and the boundary-connection pattern illustrates the detailed boundary connections to fulfill the PBCs.

Supplementary Note 3. The U(1) hyperbolic band theory of the abelian cluster with non-trivial topologies. In this part, we give a detailed discussion on the U(1) hyperbolic band theory on the finite abelian cluster with non-trivial topologies. Following the recent work about the automorphic Bloch theorems for hyperbolic lattices, we know that constructing the PBCs of the finite hyperbolic lattice should solve the normal subgroup of index N (Γ_{PBC}) in $\Gamma_{\{8,8\}}$. In this case, the (right) coset decomposition is written as

$$\Gamma_{\{8,8\}} = \Gamma_{PBC} \cup \Gamma_{PBC} g_2 \cup \dots \cup \Gamma_{PBC} g_N, \tag{5}$$

where $\cup$ denotes disjoint union, and the set of coset representatives is

$$T = \{e, g_2, \dots, g_N\} \subset \Gamma_{\{8,8\}}. \tag{6}$$

The cluster with N units (C) could be generate by the Bolza cell (D) with the group of T, that is

$$C = \mathrm{D} \cup g_2 D \cup \dots \cup g_N D. \tag{7}$$

It has been demonstrated that two units $g_i D$ and $g_j D$ are nearest neighbors on a PBC cluster if there exists a group element $\gamma_{PBC} \in \Gamma_{PBC}$ such that $\gamma_{PBC} g_j = g_i \gamma_\alpha$, where $\gamma_\alpha \in \{\gamma_1, \gamma_2, \gamma_3, \gamma_4, \gamma_1^{-1}, \gamma_2^{-1}, \gamma_3^{-1}, \gamma_4^{-1}\}$.

By Cayley's theorem, any finite group of order N admits a faithful homomorphism to the permutation group S_N. Hence, it is expected to look for the N ×N matrix representation of the group T. Here, we direct imply the conclusion proposed by Maciejko et al, that the N×N matrix representation of a group element of T is expressed as:

$$\mathcal{U}_{ij}(g_k) = \delta_{g_i, g_k g_j} \tag{8}$$

Based on the N×N matrix representation of the Supplementary equation (8), we can easily obtain matrix representations of four generators of $\Gamma_{\{8,8\}}$ with respect to the abelian clusters as

$$\mathcal{U}(\gamma_1) = \begin{bmatrix}
0&1&0&0&0&0&0&0&0&0&0&0\\
0&0&0&0&0&1&0&0&0&0&0&0\\
0&0&0&0&0&0&0&0&1&0&0&0\\
0&0&0&0&0&0&0&1&0&0&0&0\\
0&0&0&0&0&0&1&0&0&0&0&0\\
1&0&0&0&0&0&0&0&0&0&0&0\\
0&0&0&0&0&0&0&0&0&0&1&0\\
0&0&0&0&0&0&0&0&0&1&0&0\\
0&0&0&0&0&0&0&0&0&0&0&1\\
0&0&0&1&0&0&0&0&0&0&0&0\\
0&0&0&0&1&0&0&0&0&0&0&0\\
0&0&1&0&0&0&0&0&0&0&0&0
\end{bmatrix}, \mathcal{U}(\gamma_2) = \begin{bmatrix}
0&0&1&0&0&0&0&0&0&0&0&0\\
0&0&0&0&0&0&0&0&1&0&0&0\\
0&0&0&0&0&0&0&1&0&0&0&0\\
0&0&0&0&0&0&1&0&0&0&0&0\\
0&0&0&0&0&1&0&0&0&0&0&0\\
0&0&0&0&0&0&0&0&0&0&0&1\\
1&0&0&0&0&0&0&0&0&0&0&0\\
0&0&0&0&0&0&0&0&0&0&1&0\\
0&0&0&0&0&0&0&0&0&1&0&0\\
0&0&0&0&1&0&0&0&0&0&0&0\\
0&1&0&0&0&0&0&0&0&0&0&0\\
0&0&0&0&1&0&0&0&0&0&0&0
\end{bmatrix}$$

$$\mathcal{U}(\gamma_3) = \begin{bmatrix}
0&0&0&1&0&0&0&0&0&0&0&0\\
0&0&0&0&0&0&0&1&0&0&0&0\\
0&0&0&0&0&0&1&0&0&0&0&0\\
0&0&0&0&0&1&0&0&0&0&0&0\\
0&0&0&0&0&0&0&0&1&0&0&0\\
0&0&0&0&0&0&0&0&0&1&0&0\\
0&0&0&0&0&0&0&0&0&0&0&1\\
1&0&0&0&0&0&0&0&0&0&0&0\\
0&0&0&0&0&0&0&0&0&0&1&0\\
0&1&0&0&0&0&0&0&0&0&0&0\\
0&0&1&0&0&0&0&0&0&0&0&0\\
0&0&0&0&1&0&0&0&0&0&0&0
\end{bmatrix}, \mathcal{U}(\gamma_4) = \begin{bmatrix}
0&0&0&0&1&0&0&0&0&0&0&0\\
0&0&0&0&0&0&1&0&0&0&0&0\\
0&0&0&0&0&1&0&0&0&0&0&0\\
0&0&0&0&0&0&0&0&1&0&0&0\\
0&0&0&0&0&0&0&1&0&0&0&0\\
0&0&0&0&0&0&0&0&0&0&1&0\\
0&0&0&0&0&0&0&0&0&1&0&0\\
0&0&0&0&0&0&0&0&0&0&0&1\\
1&0&0&0&0&0&0&0&0&0&0&0\\
0&0&1&0&0&0&0&0&0&0&0&0\\
0&0&0&1&0&0&0&0&0&0&0&0\\
0&1&0&0&0&0&0&0&0&0&0&0
\end{bmatrix}. \tag{9}$$

Similarly, the matrix representations of four generators of $\Gamma_{\{8,8\}}$ with respect to the non-abelian clusters can be expressed as:

$$\mathcal{U}(\gamma_1) = \begin{bmatrix}
0&1&0&0&0&0&0&0&0&0&0&0\\
0&0&0&0&0&1&0&0&0&0&0&0\\
0&0&0&0&0&0&0&0&1&0&0&0\\
0&0&0&0&0&0&1&0&0&0&0&0\\
0&0&0&0&0&0&0&1&0&0&0&0\\
1&0&0&0&0&0&0&0&0&0&0&0\\
0&0&0&0&0&0&0&0&0&1&0&0\\
0&0&0&0&0&0&0&0&0&0&0&1\\
0&0&0&0&0&0&0&0&0&0&1&0\\
0&0&0&1&0&0&0&0&0&0&0&0\\
0&0&1&0&0&0&0&0&0&0&0&0\\
0&0&0&0&1&0&0&0&0&0&0&0
\end{bmatrix}, \mathcal{U}(\gamma_2) = \begin{bmatrix}
0&0&1&0&0&0&0&0&0&0&0&0\\
0&0&0&0&0&0&0&1&0&0&0&0\\
0&0&0&0&0&0&1&0&0&0&0&0\\
0&0&0&0&0&0&0&0&1&0&0&0\\
0&0&0&0&0&1&0&0&0&0&0&0\\
0&0&0&0&0&0&0&0&0&1&0&0\\
1&0&0&0&0&0&0&0&0&0&0&0\\
0&0&0&0&0&0&0&0&0&0&1&0\\
0&0&0&0&0&0&0&0&0&0&0&1\\
0&0&0&0&1&0&0&0&0&0&0&0\\
0&1&0&0&0&0&0&0&0&0&0&0\\
0&0&0&1&0&0&0&0&0&0&0&0
\end{bmatrix}$$

$$\mathcal{U}(\gamma_3) = \begin{bmatrix} 0&0&0&1&0&0&0&0&0&0&0&0 \\ 0&0&0&0&0&0&0&0&1&0&0&0 \\ 0&0&0&0&0&1&0&0&0&0&0&0 \\ 0&0&0&0&0&0&0&1&0&0&0&0 \\ 0&0&0&0&0&0&1&0&0&0&0&0 \\ 0&0&0&0&0&0&0&0&0&0&0&1 \\ 0&0&0&0&0&0&0&0&0&0&1&0 \\ 1&0&0&0&0&0&0&0&0&0&0&0 \\ 0&0&0&0&0&0&0&0&0&1&0&0 \\ 0&1&0&0&0&0&0&0&0&0&0&0 \\ 0&0&0&0&1&0&0&0&0&0&0&0 \\ 0&0&1&0&0&0&0&0&0&0&0&0 \end{bmatrix}, \mathcal{U}(\gamma_4) = \begin{bmatrix} 0&0&0&0&1&0&0&0&0&0&0&0 \\ 0&0&0&0&0&0&1&0&0&0&0&0 \\ 0&0&0&0&0&0&0&1&0&0&0&0 \\ 0&0&0&0&0&1&0&0&0&0&0&0 \\ 0&0&0&0&0&0&0&0&1&0&0&0 \\ 0&0&0&0&0&0&0&0&0&0&1&0 \\ 0&0&0&0&0&0&0&0&0&0&0&1 \\ 0&0&0&0&0&0&0&0&0&1&0&0 \\ 1&0&0&0&0&0&0&0&0&0&0&0 \\ 0&0&1&0&0&0&0&0&0&0&0&0 \\ 0&0&0&1&0&0&0&0&0&0&0&0 \\ 0&1&0&0&0&0&0&0&0&0&0&0 \end{bmatrix}. \quad (10)$$

Based on the matrix representations, it is straightforwardly to know that the four matrix representations of abelian cluster (Supplementary equation (9)) satisfy the relationship of $\mathcal{U}(\gamma_i)\mathcal{U}(\gamma_j) = \mathcal{U}(\gamma_j)\mathcal{U}(\gamma_i)$. While, as for that of non-abelian cluster, these four matrix representations (Supplementary equation (6)) obey $\mathcal{U}(\gamma_i)\mathcal{U}(\gamma_j) \neq \mathcal{U}(\gamma_j)\mathcal{U}(\gamma_i)$. These results clearly prove the abelian and non-abelian nature of our proposed hyperbolic clusters.

For a finite abelian PBC cluster, it has been demonstrated that the hyperbolic crystal momentum becomes discretized. The discretized k-vector in 4D BZ can be obtain by simultaneous diagonalization of the translation matrices of four group generators, as expressed in Supplementary equation (9). In this case, the N allowed k values correspond to N eigenvalues of these four simultaneously diagonalized matrices. Substituting the obtained k vectors into the Bloch Hamiltonian of the non-trivial hyperbolic model, we can calculate the eigen-spectra of the abelian cluster, as shown in Figures 2a and 2b of main text.

Supplementary Note 4. Numerical results of the eigen-spectra for non-abelian clusters with PBCs. In this part, we numerically calculate the eigen-spectra of finite non-abelian cluster with PBCs by the direct diagonalization. The calculated eigen-spectra with different mass terms (m=0.7, a=0.2) and (m=0.7, a=3.2) are shown in Supplementary Figures 2a and 2b, respectively. Comparing to counterparts of the abelian cluster, we can see that the significant difference of energy spectra exists between abelian and non-abelian clusters with different mass terms. This indicates that the non-abelian cluster could not be described by the $U(1)$ hyperbolic band theory of the topological Bolza cell.

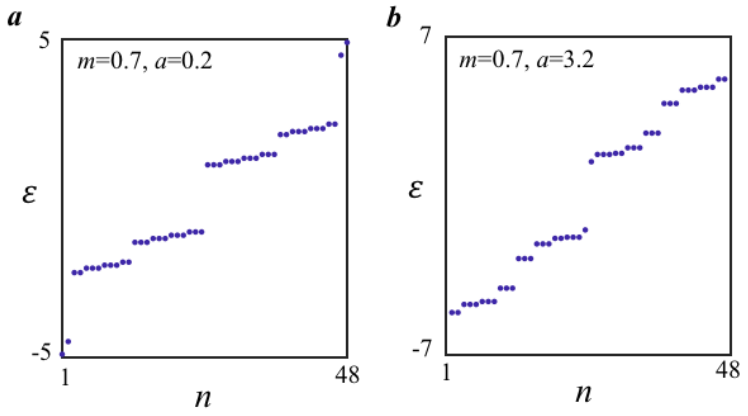

Supplementary Figure 2. Numerical results of the eigen-spectra for non-abelian clusters with PBCs. The calculated eigen-spectra of periodic non-abelian cluster with the mass term being (m=0.7, a=0.2) for (a) and (m=0.7, a=3.2) for (b).

Supplementary Note 5. Numerical results of the eigen-spectra for abelian clusters with fully OBCs. In this part, we numerically calculate the eigen-spectra of finite abelian cluster with fully OBCs. The calculated eigen-spectra with different mass terms (m=0.7, a=0.2) and (m=0.7, a=3.2) are shown in Supplementary Figures 3a and 3b, respectively. The colormap corresponds to the quantity $V(\varepsilon)$, which quantifies the localization degree of each eigenmode on boundary sites. We can see that much of eigen-modes exhibit significant boundary localizations, making the bulk modes deviate from that of the periodic hyperbolic cluster. Hence, the nontrivial boundary states are hard to be resolved.

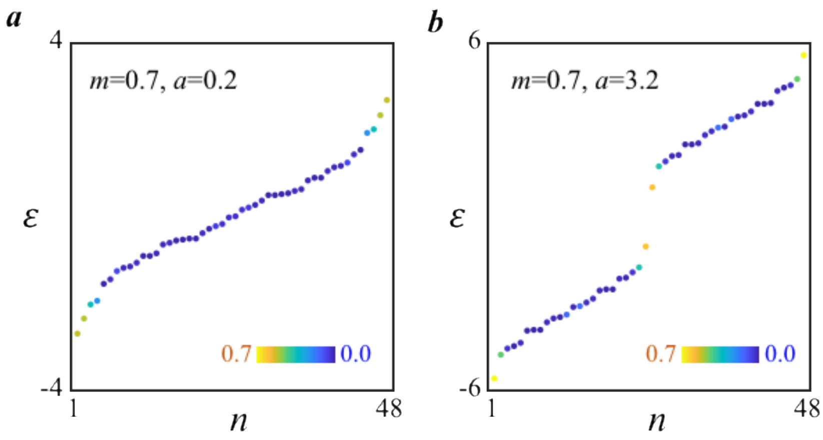

Supplementary Figure 3. Numerical results of the eigen-spectra for abelian clusters with fully OBCs. The calculated eigen-spectra with the mass term being (m=0.7, a=0.2), for (a) and (m=0.7, a=3.2) for (b).

Supplementary Note 6. Details for the partially OBCs of abelian clusters. In this part, we illustrate the partially OBCs of abelian clusters. As shown in Supplementary Figure 4, the inter-cell couplings between boundary units marked by red are changing from PBCs to OBCs. Specifically, the inter-cell coupling between boundary units marked by letters of ‘*i*’, ‘*l*’, ‘*e*’, ‘*b*’, ‘*c*’, ‘*d*’ are deleted to construct partially OBCs of abelian clusters. In this case, the boundary states in the non-trivial band gap are localized around these units with open boundaries, as shown in Figs. 2g and 2h in the main text.

γ_1	e to g	j to d	f to a	k to e	g to k	h to j	i to l	l to c
γ_2	e to f	j to e	f to l	k to b	g to a	h to k	i to j	l to d
γ_3	e to i	j to b	f to j	k to c	g to l	h to a	i to k	l to e
γ_4	e to h	j to c	f to k	k to d	g to j	h to l	i to a	l to b
γ_1^{-1}	e to k	j to h	f to b	k to g	g to e	h to d	i to c	l to i
γ_2^{-1}	e to j	j to i	f to e	k to h	g to d	h to c	i to b	l to f
γ_3^{-1}	e to l	j to f	f to d	k to i	g to c	h to b	i to e	l to g
γ_4^{-1}	e to a	j to d	f to c	k to f	g to b	h to e	i to d	l to h

Supplementary Figure 4. Details for the partially OBCs of abelian clusters.

Supplementary Note 7. Details for the derivation of eigenequations for hyperbolic circuits. In this section, we give a detailed derivation of the circuit eigenequation and the correspondence between the designed hyperbolic circuit and hyperbolic 4D quantum Hall model. Here, each lattice site possesses four circuit nodes. In this case, the voltage and current at the circuit node, which corresponds to the *j*-site in the *i*-unit, should be written as $V_i = [V_{i,j,1}, V_{i,j,2}, V_{i,j,3}, V_{i,j,4}]^T$ and $I_i = [I_{i,j,1}, I_{i,j,2}, I_{i,j,3}, I_{i,j,4}]^T$, respectively. Here, four lattice sites with the mass term being *m*-*a*, *m*+*a*, -*m*+*a* and -*m*-*a* in a single unit are labeled by 1-site, 2-site, 3-site, and 4-site, respectively. The voltage on circuit node is in the form of $V_{i,j,(1,2,3,4)}e^{i\omega t}$. Firstly, we focus on four nodes, which work as a single lattice site in the *i*-unit, located in the bulk region of the hyperbolic model with the mass term being *m*-*a*. Carrying out Kirchhoff's law on four circuit nodes, we obtain the following equation:

$$\begin{bmatrix} I_{i,1,1} \\ I_{i,1,2} \\ I_{i,1,3} \\ I_{i,1,4} \end{bmatrix} = i\omega^{-1}(\omega^2 C \begin{bmatrix} 2 & -1 & 0 & -1 \\ -1 & 2 & -1 & 0 \\ 0 & -1 & 2 & -1 \\ -1 & 0 & -1 & 2 \end{bmatrix} \begin{bmatrix} V_{i,1,1} \\ V_{i,1,2} \\ V_{i,1,3} \\ V_{i,1,4} \end{bmatrix} + \omega^2 C_J \begin{bmatrix} V_{i,1,1} - V_{i+\gamma_1,1,3} \\ V_{i,1,2} - V_{i+\gamma_1,1,4} \\ V_{i,1,3} - V_{i+\gamma_1,1,1} \\ V_{i,1,4} - V_{i+\gamma_1,1,2} \end{bmatrix} + \omega^2 C_t \begin{bmatrix} V_{i,1,1} - V_{i+\gamma_1,3,3} \\ V_{i,1,2} - V_{i+\gamma_1,3,4} \\ V_{i,1,3} - V_{i+\gamma_1,3,1} \\ V_{i,1,4} - V_{i+\gamma_1,3,2} \end{bmatrix}$$

$$
+\omega^2 C_J\begin{bmatrix} V_{i,1,1}-V_{i+\gamma_1^{-1},1,3} \\ V_{i,1,2}-V_{i+\gamma_1^{-1},1,4} \\ V_{i,1,3}-V_{i+\gamma_1^{-1},1,1} \\ V_{i,1,4}-V_{i+\gamma_1^{-1},1,2} \end{bmatrix} + \omega^2 C_t\begin{bmatrix} V_{i,1,1}-V_{i+\gamma_1^{-1},3,1} \\ V_{i,1,2}-V_{i+\gamma_1^{-1},3,2} \\ V_{i,1,3}-V_{i+\gamma_1^{-1},3,3} \\ V_{i,1,4}-V_{i+\gamma_1^{-1},3,4} \end{bmatrix} + \omega^2 C_J\begin{bmatrix} V_{i,1,1}-V_{i+\gamma_2,1,3} \\ V_{i,1,2}-V_{i+\gamma_2,1,4} \\ V_{i,1,3}-V_{i+\gamma_2,1,1} \\ V_{i,1,4}-V_{i+\gamma_2,1,2} \end{bmatrix} + \omega^2 C_t\begin{bmatrix} V_{i,1,1}-V_{i+\gamma_2,4,2} \\ V_{i,1,2}-V_{i+\gamma_2,4,3} \\ V_{i,1,3}-V_{i+\gamma_2,4,4} \\ V_{i,1,4}-V_{i+\gamma_2,4,1} \end{bmatrix}
$$

$$
+\omega^2 C_J\begin{bmatrix} V_{i,1,1}-V_{i+\gamma_2^{-1},1,3} \\ V_{i,1,2}-V_{i+\gamma_2^{-1},1,4} \\ V_{i,1,3}-V_{i+\gamma_2^{-1},1,1} \\ V_{i,1,4}-V_{i+\gamma_2^{-1},1,2} \end{bmatrix} + \omega^2 C_t\begin{bmatrix} V_{i,1,1}-V_{i+\gamma_2^{-1},4,4} \\ V_{i,1,2}-V_{i+\gamma_2^{-1},4,1} \\ V_{i,1,3}-V_{i+\gamma_2^{-1},4,2} \\ V_{i,1,4}-V_{i+\gamma_2^{-1},4,3} \end{bmatrix} + \omega^2 C_J\begin{bmatrix} V_{i,1,1}-V_{i+\gamma_3,1,3} \\ V_{i,1,2}-V_{i+\gamma_3,1,4} \\ V_{i,1,3}-V_{i+\gamma_3,1,1} \\ V_{i,1,4}-V_{i+\gamma_3,1,2} \end{bmatrix} + \omega^2 C_t\begin{bmatrix} V_{i,1,1}-V_{i+\gamma_3,4,1} \\ V_{i,1,2}-V_{i+\gamma_3,4,2} \\ V_{i,1,3}-V_{i+\gamma_3,4,3} \\ V_{i,1,4}-V_{i+\gamma_3,4,4} \end{bmatrix}
$$

$$
+\omega^2 C_J\begin{bmatrix} V_{i,1,1}-V_{i+\gamma_3^{-1},1,3} \\ V_{i,1,2}-V_{i+\gamma_3^{-1},1,4} \\ V_{i,1,3}-V_{i+\gamma_3^{-1},1,1} \\ V_{i,1,4}-V_{i+\gamma_3^{-1},1,2} \end{bmatrix} + \omega^2 C_t\begin{bmatrix} V_{i,1,1}-V_{i+\gamma_3^{-1},4,3} \\ V_{i,1,2}-V_{i+\gamma_3^{-1},4,4} \\ V_{i,1,3}-V_{i+\gamma_3^{-1},4,1} \\ V_{i,1,4}-V_{i+\gamma_3^{-1},4,2} \end{bmatrix} + \omega^2 C_J\begin{bmatrix} V_{i,1,1}-V_{i+\gamma_4,1,3} \\ V_{i,1,2}-V_{i+\gamma_4,1,4} \\ V_{i,1,3}-V_{i+\gamma_4,1,1} \\ V_{i,1,4}-V_{i+\gamma_4,1,2} \end{bmatrix} + \omega^2 C_t\begin{bmatrix} V_{i,1,1}-V_{i+\gamma_4,3,2} \\ V_{i,1,2}-V_{i+\gamma_4,3,3} \\ V_{i,1,3}-V_{i+\gamma_4,3,4} \\ V_{i,1,4}-V_{i+\gamma_4,3,1} \end{bmatrix}
$$

$$
+\omega^2 C_J\begin{bmatrix} V_{i,1,1}-V_{i+\gamma_4^{-1},1,3} \\ V_{i,1,2}-V_{i+\gamma_4^{-1},1,4} \\ V_{i,1,3}-V_{i+\gamma_4^{-1},1,1} \\ V_{i,1,4}-V_{i+\gamma_4^{-1},1,2} \end{bmatrix} + \omega^2 C_t\begin{bmatrix} V_{i,1,1}-V_{i+\gamma_4^{-1},3,4} \\ V_{i,1,2}-V_{i+\gamma_4^{-1},3,1} \\ V_{i,1,3}-V_{i+\gamma_4^{-1},3,2} \\ V_{i,1,4}-V_{i+\gamma_4^{-1},3,3} \end{bmatrix} + \omega^2(m-a)C_g\begin{bmatrix} V_{i,1,1} \\ V_{i,1,2} \\ V_{i,1,3} \\ V_{i,1,4} \end{bmatrix} + \omega^2 C_u\begin{bmatrix} V_{i,1,1} \\ V_{i,1,2} \\ V_{i,1,3} \\ V_{i,1,4} \end{bmatrix} - \frac{1}{L_g}\begin{bmatrix} V_{i,1,1} \\ V_{i,1,2} \\ V_{i,1,3} \\ V_{i,1,4} \end{bmatrix} \quad (11)
$$

where C_J and C_t are capacitances corresponding to intercell coupling strength of $J_{1,2,3,4}$ and $t_{1,2,3,4}$. C is the capacitance used for connecting circuit nodes belonging to the same site. L_g is the inductor linking the circuit nodes to the ground. $(m-a)C_g$ is grounding capacitance corresponding to the onsite potential of *m-a*. C_u is the capacitance related to the homogeneous onsite potential. We assume that there is no external source, so that the current flowing out of the node is zero. In this case, Supplementary Eq. (11) becomes:

$$
\frac{1}{\omega^2 L_g}\begin{bmatrix} V_{i,1,1} \\ V_{i,1,2} \\ V_{i,1,3} \\ V_{i,1,4} \end{bmatrix} = C\begin{bmatrix} 2 & -1 & 0 & -1 \\ -1 & 2 & -1 & 0 \\ 0 & -1 & 2 & -1 \\ -1 & 0 & -1 & 2 \end{bmatrix}\begin{bmatrix} V_{i,1,1} \\ V_{i,1,2} \\ V_{i,1,3} \\ V_{i,1,4} \end{bmatrix} + (8C_J + 8C_t + (m-a)C_g + C_u)\begin{bmatrix} V_{i,1,1} \\ V_{i,1,2} \\ V_{i,1,3} \\ V_{i,1,4} \end{bmatrix} - C_J\begin{bmatrix} V_{i+\gamma_1,1,3} \\ V_{i+\gamma_1,1,4} \\ V_{i+\gamma_1,1,1} \\ V_{i+\gamma_1,1,2} \end{bmatrix}
$$

$$
-C_t\begin{bmatrix} V_{i+\gamma_1,3,3} \\ V_{i+\gamma_1,3,4} \\ V_{i+\gamma_1,3,1} \\ V_{i+\gamma_1,3,2} \end{bmatrix} - C_J\begin{bmatrix} V_{i+\gamma_1^{-1},1,3} \\ V_{i+\gamma_1^{-1},1,4} \\ V_{i+\gamma_1^{-1},1,1} \\ V_{i+\gamma_1^{-1},1,2} \end{bmatrix} - C_t\begin{bmatrix} V_{i+\gamma_1^{-1},3,1} \\ V_{i+\gamma_1^{-1},3,2} \\ V_{i+\gamma_1^{-1},3,3} \\ V_{i+\gamma_1^{-1},3,4} \end{bmatrix} - C_J\begin{bmatrix} V_{i+\gamma_2,1,3} \\ V_{i+\gamma_2,1,4} \\ V_{i+\gamma_2,1,1} \\ V_{i+\gamma_2,1,2} \end{bmatrix} - C_t\begin{bmatrix} V_{i+\gamma_2,4,2} \\ V_{i+\gamma_2,4,3} \\ V_{i+\gamma_2,4,4} \\ V_{i+\gamma_2,4,1} \end{bmatrix} - C_J\begin{bmatrix} V_{i+\gamma_2^{-1},1,3} \\ V_{i+\gamma_2^{-1},1,4} \\ V_{i+\gamma_2^{-1},1,1} \\ V_{i+\gamma_2^{-1},1,2} \end{bmatrix}
$$

$$
-C_t\begin{bmatrix} V_{i+\gamma_2^{-1},4,4} \\ V_{i+\gamma_2^{-1},4,1} \\ V_{i+\gamma_2^{-1},4,2} \\ V_{i+\gamma_2^{-1},4,3} \end{bmatrix} - C_J\begin{bmatrix} V_{i+\gamma_3,1,3} \\ V_{i+\gamma_3,1,4} \\ V_{i+\gamma_3,1,1} \\ V_{i+\gamma_3,1,2} \end{bmatrix} - C_t\begin{bmatrix} V_{i+\gamma_3,4,2} \\ V_{i+\gamma_3,4,3} \\ V_{i+\gamma_3,4,4} \\ V_{i+\gamma_3,4,1} \end{bmatrix} - C_J\begin{bmatrix} V_{i+\gamma_3^{-1},1,3} \\ V_{i+\gamma_3^{-1},1,4} \\ V_{i+\gamma_3^{-1},1,1} \\ V_{i+\gamma_3^{-1},1,2} \end{bmatrix} - C_t\begin{bmatrix} V_{i+\gamma_3^{-1},4,3} \\ V_{i+\gamma_3^{-1},4,4} \\ V_{i+\gamma_3^{-1},4,1} \\ V_{i+\gamma_3^{-1},4,2} \end{bmatrix} - C_J\begin{bmatrix} V_{i+\gamma_4,1,3} \\ V_{i+\gamma_4,1,4} \\ V_{i+\gamma_4,1,1} \\ V_{i+\gamma_4,1,2} \end{bmatrix}
$$

$$
-C_t\begin{bmatrix} V_{i+\gamma_4,3,2} \\ V_{i+\gamma_4,3,3} \\ V_{i+\gamma_4,3,4} \\ V_{i+\gamma_4,3,1} \end{bmatrix} - C_J\begin{bmatrix} V_{i+\gamma_4^{-1},1,3} \\ V_{i+\gamma_4^{-1},1,4} \\ V_{i+\gamma_4^{-1},1,1} \\ V_{i+\gamma_4^{-1},1,2} \end{bmatrix} - C_t\begin{bmatrix} V_{i+\gamma_4^{-1},3,4} \\ V_{i+\gamma_4^{-1},3,1} \\ V_{i+\gamma_4^{-1},3,2} \\ V_{i+\gamma_4^{-1},3,3} \end{bmatrix} \quad (12)
$$

Performing the diagonalization of Supplementary Eq. (12) with a unitary transformation:

$$F=\frac{1}{\sqrt{4}}\begin{bmatrix}1 & 1 & 1 & 1\\ 1 & e^{i2\pi/4} & e^{i4\pi/4} & e^{i6\pi/4}\\ 1 & e^{i4\pi/4} & e^{i8\pi/4} & e^{i12\pi/4}\\ 1 & e^{i6\pi/4} & e^{i12\pi/4} & e^{i18\pi/4}\end{bmatrix}. \tag{13}$$

Supplementary Eq. (12) becomes:

$$\frac{1}{\omega^2 L_g}\begin{bmatrix}V_{i,1,\rightarrow}\\ V_{i,1,\uparrow}\\ V_{i,1,\leftarrow}\\ V_{i,1,\downarrow}\end{bmatrix}=C\begin{bmatrix}0&0&0&0\\0&2&0&0\\0&0&4&0\\0&0&0&2\end{bmatrix}\begin{bmatrix}V_{i,1,\rightarrow}\\ V_{i,1,\uparrow}\\ V_{i,1,\leftarrow}\\ V_{i,1,\downarrow}\end{bmatrix}+(8C_J+8C_t+(m-a)C_g+C_u)\begin{bmatrix}V_{i,1,\rightarrow}\\ V_{i,1,\uparrow}\\ V_{i,1,\leftarrow}\\ V_{i,1,\downarrow}\end{bmatrix}$$

$$-C_J\begin{bmatrix}1&0&0&0\\0&e^{i\pi}&0&0\\0&0&1&0\\0&0&0&e^{i\pi}\end{bmatrix}\begin{bmatrix}V_{i+\gamma_1,1,\rightarrow}\\ V_{i+\gamma_1,1,\uparrow}\\ V_{i+\gamma_1,1,\leftarrow}\\ V_{i+\gamma_1,1,\downarrow}\end{bmatrix}-C_t\begin{bmatrix}1&0&0&0\\0&e^{i\pi}&0&0\\0&0&1&0\\0&0&0&e^{i\pi}\end{bmatrix}\begin{bmatrix}V_{i+\gamma_1,3,\rightarrow}\\ V_{i+\gamma_1,3,\uparrow}\\ V_{i+\gamma_1,3,\leftarrow}\\ V_{i+\gamma_1,3,4\downarrow}\end{bmatrix}-C_J\begin{bmatrix}1&0&0&0\\0&e^{i\pi}&0&0\\0&0&1&0\\0&0&0&e^{i\pi}\end{bmatrix}\begin{bmatrix}V_{i+\gamma_1^{-1},1,\rightarrow}\\ V_{i+\gamma_1^{-1},1,\uparrow}\\ V_{i+\gamma_1^{-1},1,\leftarrow}\\ V_{i+\gamma_1^{-1},1,\downarrow}\end{bmatrix}$$

$$-C_t\begin{bmatrix}V_{i+\gamma_1^{-1},3,\rightarrow}\\ V_{i+\gamma_1^{-1},3,\uparrow}\\ V_{i+\gamma_1^{-1},3,\leftarrow}\\ V_{i+\gamma_1^{-1},3,\downarrow}\end{bmatrix}-C_J\begin{bmatrix}1&0&0&0\\0&e^{i\pi}&0&0\\0&0&1&0\\0&0&0&e^{i\pi}\end{bmatrix}\begin{bmatrix}V_{i+\gamma_2,1,\rightarrow}\\ V_{i+\gamma_2,1,\uparrow}\\ V_{i+\gamma_2,1,\leftarrow}\\ V_{i+\gamma_2,1,\downarrow}\end{bmatrix}-C_t\begin{bmatrix}1&0&0&0\\0&e^{i\pi/2}&0&0\\0&0&e^{i\pi}&0\\0&0&0&e^{-i\pi/2}\end{bmatrix}\begin{bmatrix}V_{i+\gamma_2,4,\rightarrow}\\ V_{i+\gamma_2,4,\uparrow}\\ V_{i+\gamma_2,4,\leftarrow}\\ V_{i+\gamma_2,4,\downarrow}\end{bmatrix}$$

$$-C_J\begin{bmatrix}1&0&0&0\\0&e^{i\pi}&0&0\\0&0&1&0\\0&0&0&e^{i\pi}\end{bmatrix}\begin{bmatrix}V_{i+\gamma_2^{-1},1,\rightarrow}\\ V_{i+\gamma_2^{-1},1,\uparrow}\\ V_{i+\gamma_2^{-1},1,\leftarrow}\\ V_{i+\gamma_2^{-1},1,\downarrow}\end{bmatrix}-C_t\begin{bmatrix}1&0&0&0\\0&e^{-i\pi/2}&0&0\\0&0&e^{i\pi}&0\\0&0&0&e^{i\pi/2}\end{bmatrix}\begin{bmatrix}V_{i+\gamma_2^{-1},4,\rightarrow}\\ V_{i+\gamma_2^{-1},4,\uparrow}\\ V_{i+\gamma_2^{-1},4,\leftarrow}\\ V_{i+\gamma_2^{-1},4,\downarrow}\end{bmatrix}$$

$$-C_J\begin{bmatrix}1&0&0&0\\0&e^{i\pi}&0&0\\0&0&1&0\\0&0&0&e^{i\pi}\end{bmatrix}\begin{bmatrix}V_{i+\gamma_3,1,\rightarrow}\\ V_{i+\gamma_3,1,\uparrow}\\ V_{i+\gamma_3,1,\leftarrow}\\ V_{i+\gamma_3,1,\downarrow}\end{bmatrix}-C_t\begin{bmatrix}V_{i+\gamma_3,4,\rightarrow}\\ V_{i+\gamma_3,4,\uparrow}\\ V_{i+\gamma_3,4,\leftarrow}\\ V_{i+\gamma_3,4,\downarrow}\end{bmatrix}-C_J\begin{bmatrix}1&0&0&0\\0&e^{i\pi}&0&0\\0&0&1&0\\0&0&0&e^{i\pi}\end{bmatrix}\begin{bmatrix}V_{i+\gamma_3^{-1},1,\rightarrow}\\ V_{i+\gamma_3^{-1},1,\uparrow}\\ V_{i+\gamma_3^{-1},1,\leftarrow}\\ V_{i+\gamma_3^{-1},1,\downarrow}\end{bmatrix}$$

$$-C_t\begin{bmatrix}1&0&0&0\\0&e^{i\pi}&0&0\\0&0&1&0\\0&0&0&e^{i\pi}\end{bmatrix}\begin{bmatrix}V_{i+\gamma_3^{-1},4,\rightarrow}\\ V_{i+\gamma_3^{-1},4,\uparrow}\\ V_{i+\gamma_3^{-1},4,\leftarrow}\\ V_{i+\gamma_3^{-1},4,\downarrow}\end{bmatrix}-C_J\begin{bmatrix}1&0&0&0\\0&e^{i\pi}&0&0\\0&0&1&0\\0&0&0&e^{i\pi}\end{bmatrix}\begin{bmatrix}V_{i+\gamma_4,1,\rightarrow}\\ V_{i+\gamma_4,1,\uparrow}\\ V_{i+\gamma_4,1,\leftarrow}\\ V_{i+\gamma_4,1,\downarrow}\end{bmatrix}-C_t\begin{bmatrix}1&0&0&0\\0&e^{i\pi/2}&0&0\\0&0&e^{i\pi}&0\\0&0&0&e^{-i\pi/2}\end{bmatrix}\begin{bmatrix}V_{i+\gamma_4,3,\rightarrow}\\ V_{i+\gamma_4,3,\uparrow}\\ V_{i+\gamma_4,3,\leftarrow}\\ V_{i+\gamma_4,3,\downarrow}\end{bmatrix}$$

$$-C_J\begin{bmatrix}1&0&0&0\\0&e^{i\pi}&0&0\\0&0&1&0\\0&0&0&e^{i\pi}\end{bmatrix}\begin{bmatrix}V_{i+\gamma_4^{-1},1,\rightarrow}\\ V_{i+\gamma_4^{-1},1,\uparrow}\\ V_{i+\gamma_4^{-1},1,\leftarrow}\\ V_{i+\gamma_4^{-1},1,\downarrow}\end{bmatrix}-C_t\begin{bmatrix}1&0&0&0\\0&e^{-i\pi/2}&0&0\\0&0&e^{i\pi}&0\\0&0&0&e^{i\pi/2}\end{bmatrix}\begin{bmatrix}V_{i+\gamma_4^{-1},3,\rightarrow}\\ V_{i+\gamma_4^{-1},3,\uparrow}\\ V_{i+\gamma_4^{-1},3,\leftarrow}\\ V_{i+\gamma_4^{-1},3,\downarrow}\end{bmatrix} \tag{14}$$

The new basis is $V_{i,1,(\rightarrow,\uparrow,\leftarrow,\downarrow)}=F[V_{i,1,1},V_{i,1,2},V_{i,1,3},V_{i,1,4}]^T$, which are four decoupled terms and two frequency-dependent terms $V_{i,1,(\uparrow,\downarrow)}$ acting as a pair of pseudospins $V_{i,1,\uparrow}=V_{i,1,1}+V_{i,1,2}e^{i\pi/2}+V_{i,1,3}e^{i\pi}+V_{i,1,4}e^{-i\pi/2}$ and $V_{i,1,\downarrow}=V_{i,1,1}+V_{i,1,2}e^{-i\pi/2}+V_{i,1,3}e^{i\pi}+V_{i,1,4}e^{i\pi/2}$. Thus, the eigen-equation of the pseudospins $V_{i,1,\uparrow}$ can be expressed as:

$$\left\{\frac{1}{\omega^2 L_g C}-2-\frac{8C_J+8C_t+C_u}{C}\right\}V_{i,1,\uparrow}=-e^{i\pi}\frac{C_J}{C}V_{i+\gamma_1,1,\uparrow}-e^{i\pi}\frac{C_t}{C}V_{i+\gamma_1,3,\uparrow}-e^{i\pi}\frac{C_J}{C}V_{i+\gamma_1^{-1},1,\uparrow}-\frac{C_t}{C}V_{i+\gamma_1^{-1},3,\uparrow}$$

$$-e^{i\pi}\frac{C_J}{C}V_{i+\gamma_2,1,\uparrow}-e^{i\pi/2}\frac{C_t}{C}V_{i+\gamma_2,4,\uparrow}-e^{i\pi}\frac{C_J}{C}V_{i+\gamma_2^{-1},1,\uparrow}-e^{-i\pi/2}\frac{C_t}{C}V_{i+\gamma_2^{-1},4,\uparrow}-e^{i\pi}\frac{C_J}{C}V_{i+\gamma_3,1,\uparrow}-\frac{C_t}{C}V_{i+\gamma_3,4,\uparrow}$$

$$-e^{i\pi}\frac{C_J}{C}V_{i+\gamma_3^{-1},1,\uparrow}-e^{i\pi}\frac{C_t}{C}V_{i+\gamma_3^{-1},4,\uparrow}-e^{i\pi}\frac{C_J}{C}V_{i+\gamma_4,1,\uparrow}-e^{i\pi/2}\frac{C_t}{C}V_{i+\gamma_4,3,\uparrow}-e^{i\pi}\frac{C_J}{C}V_{i+\gamma_4^{-1},1,\uparrow}-e^{-i\pi/2}\frac{C_t}{C}V_{i+\gamma_4^{-1},3,\uparrow}$$

$$+(m-a)\frac{C_g}{C}V_{i,1,\uparrow} \tag{15}$$

Similarly, eigen-equations for other three groups of circuit nodes corresponding to lattice sites with onsite potential being $m+a$, $-m+a$ and $-m-a$ can also be evaluated by Eq. (16) to Eq. (18)

$$\left\{\frac{1}{\omega^2 L_g C}-2-\frac{8C_J+8C_t+C_u}{C}\right\}V_{i,2,\uparrow}=-e^{i\pi}\frac{C_J}{C}V_{i+\gamma_1,2,\uparrow}-e^{i\pi}\frac{C_t}{C}V_{i+\gamma_1,4,\uparrow}-e^{i\pi}\frac{C_J}{C}V_{i+\gamma_1^{-1},2,\uparrow}-\frac{C_t}{C}V_{i+\gamma_1^{-1},4,\uparrow}$$
$$-e^{i\pi}\frac{C_J}{C}V_{i+\gamma_2,2,\uparrow}-e^{i\pi/2}\frac{C_t}{C}V_{i+\gamma_2,3,\uparrow}-e^{i\pi}\frac{C_J}{C}V_{i+\gamma_2^{-1},2,\uparrow}-e^{-i\pi/2}\frac{C_t}{C}V_{i+\gamma_2^{-1},3,\uparrow}-e^{i\pi}\frac{C_J}{C}V_{i+\gamma_3,2,\uparrow}-e^{i\pi}\frac{C_t}{C}V_{i+\gamma_3,3,\uparrow}$$
$$-e^{i\pi}\frac{C_J}{C}V_{i+\gamma_3^{-1},2,\uparrow}-\frac{C_t}{C}V_{i+\gamma_3^{-1},3,\uparrow}-e^{i\pi}\frac{C_J}{C}V_{i+\gamma_4,2,\uparrow}-e^{-i\pi/2}\frac{C_t}{C}V_{i+\gamma_4,4,\uparrow}-e^{i\pi}\frac{C_J}{C}V_{i+\gamma_4^{-1},2,\uparrow}-e^{i\pi/2}\frac{C_t}{C}V_{i+\gamma_4^{-1},4,\uparrow}$$
$$+(m+a)\frac{C_g}{C}V_{i,2,\uparrow} \tag{16}$$

$$\left\{\frac{1}{\omega^2 L_g C}-2-\frac{8C_J+8C_t+C_u}{C}\right\}V_{i,3,\uparrow}=-\frac{C_J}{C}V_{i+\gamma_1,3,\uparrow}-\frac{C_t}{C}V_{i+\gamma_1,1,\uparrow}-\frac{C_J}{C}V_{i+\gamma_1^{-1},3,\uparrow}-e^{i\pi}\frac{C_t}{C}V_{i+\gamma_1^{-1},1,\uparrow}$$
$$-\frac{C_J}{C}V_{i+\gamma_2,3,\uparrow}-e^{i\pi/2}\frac{C_t}{C}V_{i+\gamma_2,2,\uparrow}-\frac{C_J}{C}V_{i+\gamma_2^{-1},3,\uparrow}-e^{-i\pi/2}\frac{C_t}{C}V_{i+\gamma_2^{-1},2,\uparrow}-\frac{C_J}{C}V_{i+\gamma_3,3,\uparrow}-\frac{C_t}{C}V_{i+\gamma_3,2,\uparrow}$$
$$-\frac{C_J}{C}V_{i+\gamma_3^{-1},3,\uparrow}-e^{i\pi}\frac{C_t}{C}V_{i+\gamma_3^{-1},2,\uparrow}-\frac{C_J}{C}V_{i+\gamma_4,3,\uparrow}-e^{i\pi/2}\frac{C_t}{C}V_{i+\gamma_4,1,\uparrow}-\frac{C_J}{C}V_{i+\gamma_4^{-1},3,\uparrow}-e^{-i\pi/2}\frac{C_t}{C}V_{i+\gamma_4^{-1},1,\uparrow}$$
$$+(-m+a)\frac{C_g}{C}V_{i,3,\uparrow} \tag{17}$$

$$\left\{\frac{1}{\omega^2 L_g C}-2-\frac{8C_J+8C_t+C_u}{C}\right\}V_{i,4,\uparrow}=-\frac{C_J}{C}V_{i+\gamma_1,4,\uparrow}-\frac{C_t}{C}V_{i+\gamma_1,2,\uparrow}-\frac{C_J}{C}V_{i+\gamma_1^{-1},4,\uparrow}-e^{i\pi}\frac{C_t}{C}V_{i+\gamma_1^{-1},2,\uparrow}$$
$$-\frac{C_J}{C}V_{i+\gamma_2,4,\uparrow}-e^{i\pi/2}\frac{C_t}{C}V_{i+\gamma_2,1,\uparrow}-\frac{C_J}{C}V_{i+\gamma_2^{-1},4,\uparrow}-e^{-i\pi/2}\frac{C_t}{C}V_{i+\gamma_2^{-1},1,\uparrow}-\frac{C_J}{C}V_{i+\gamma_3,4,\uparrow}-e^{i\pi}\frac{C_t}{C}V_{i+\gamma_3,1,\uparrow}$$
$$-\frac{C_J}{C}V_{i+\gamma_3^{-1},4,\uparrow}-\frac{C_t}{C}V_{i+\gamma_3^{-1},1,\uparrow}-\frac{C_J}{C}V_{i+\gamma_4,4,\uparrow}-e^{-i\pi/2}\frac{C_t}{C}V_{i+\gamma_4,2,\uparrow}-\frac{C_J}{C}V_{i+\gamma_4^{-1},4,\uparrow}-e^{i\pi/2}\frac{C_t}{C}V_{i+\gamma_4^{-1},2,\uparrow}$$
$$+(-m-a)\frac{C_g}{C}V_{i,4,\uparrow} \tag{18}$$

In this case, we provide the following identification of tight-binding parameters in terms of circuit elements:

$$t_{1,2,3,4}=\frac{C_{\mathrm{t}}}{C}, J_{1,2,3,4}=\frac{C_J}{C}, \varepsilon=\frac{f_0{}^2}{f^2}-2-\frac{8C_J+8C_t+C_u}{C}, m=m\frac{C_g}{C}, a=a\frac{C_g}{C},\ f_0=\frac{1}{2\pi\sqrt{CL}}, \tag{19}$$

We note that Eqs. (15)-(18) can be written in the form of $\boldsymbol{I}=J\boldsymbol{V}$ with J being the circuit Laplacian, $\boldsymbol{I}=0$ being the input current, and $\boldsymbol{V}$ being the voltage pseudospins. In this case, the Hamiltonian of the associated hyperbolic lattice model can be written as $H=(\frac{1}{\omega^2 L_g C}-2-\frac{8C_J+8C_t+C_u}{C})*\mathbb{I}-J$ with $\mathbb{I}$ being an identical matrix.

Supplementary Note 8. The influence of lossy effects on impedance responses. In this part, we numerically investigated the influence of lossy effects on the impedance responses of hyperbolic circuit networks. To quantitatively estimate the loss of our circuit samples, we calculate the impedance responses of the designed hyperbolic Chern circuit with the effective series resistances of inductance

being 50 $m\Omega$, 150 $m\Omega$, and 300 $m\Omega$. Simulation results in Supplementary Figures 5a and 5b correspond to the circuit with parameters identical to Figs. 3e and 4a, respectively. And, simulation results in Supplementary Figures 5c and 5d correspond to the circuit with parameters identical to Figs. 3f and 4b, respectively. It is shown that with the series resistances of inductance being increased, the impedance peaks of both bulk and boundary nodes are broadening.

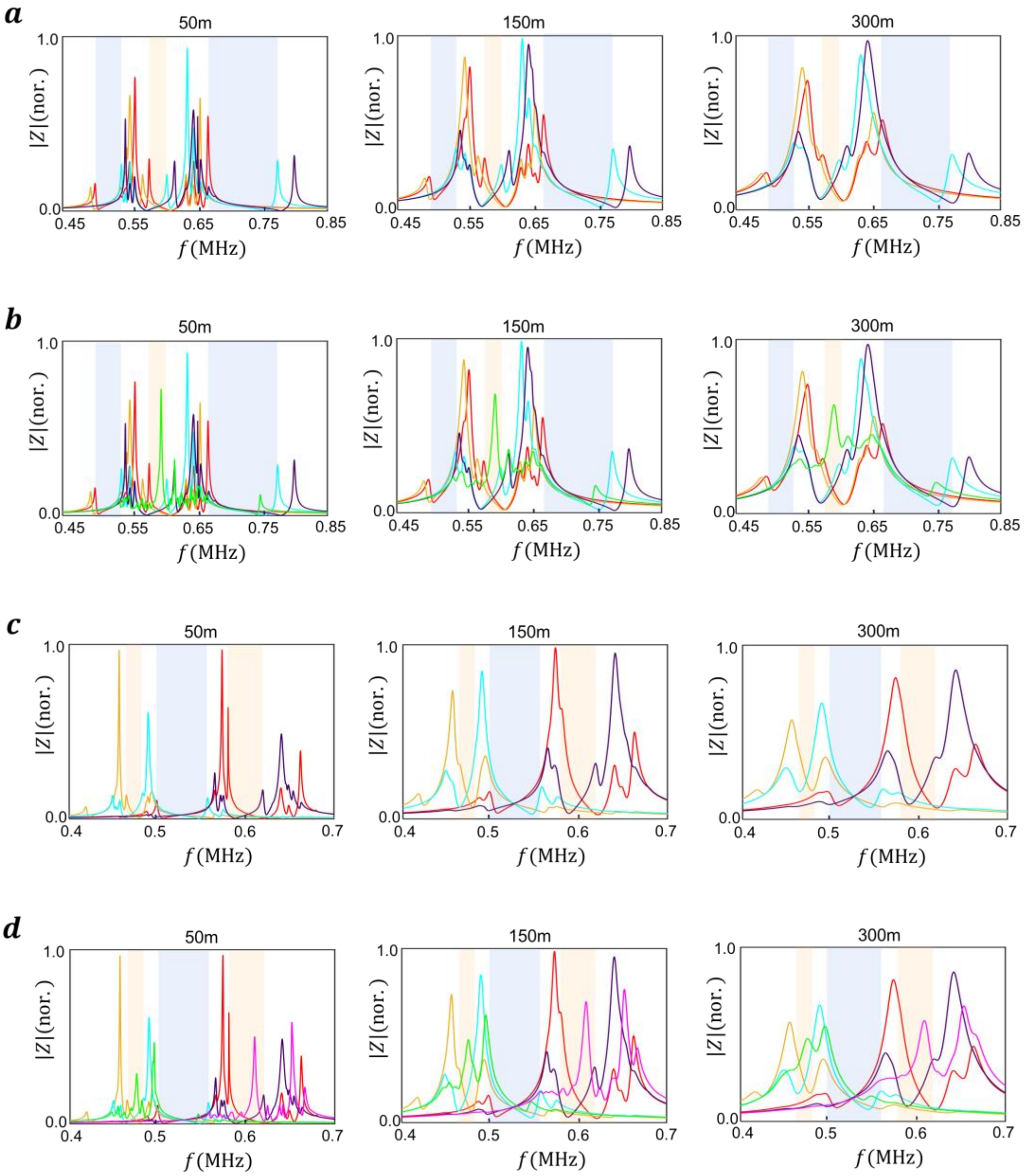

Supplementary Figure 5. Calculated impedance responses of hyperbolic circuits with different effective series resistances of inductance. (a) and (c). The simulated impedance responses of bulk nodes in periodic circuit with different losses, and the mass terms is (*m*=0.7, *a*=0.2) and (*m*=0.7, *a*=3.2), respectively. (b) and (d). The simulated impedance responses of bulk and boundary nodes in circuits possessing different losses under partially OBCs, and the mass terms is (*m*=0.7, *a*=0.2) and (*m*=0.7, *a*=3.2), respectively.
